# Genome-wide analysis exploring mechanisms used by *Shigella sonnei* to survive long-term nutrient starvation

**DOI:** 10.1128/msystems.00088-26

**Published:** 2026-03-16

**Authors:** Xosé M. Matanza, P. Brian Leung, Vincenzo Torraca, Jayne Watson, Matthew J. Dorman, Nicholas R. Thomson, Abigail Clements

**Affiliations:** 1 Centre for Bacterial Resistance Biology, Department of Life Sciences, Imperial College London, South Kensington Campus 4615, London, United Kingdom; 2 Department of Infectious Diseases, School of Immunology and Microbial Sciences, King's College London 228083, London, United Kingdom; 3 Wellcome Sanger Institute, Wellcome Genome Campus 47665, Hinxton, United Kingdom; University of California Irvine, Irvine, California, USA

**Keywords:** *Shigella*, nutrient starvation, Tn-seq, microbial metabolism, bacterial cell envelope

## Abstract

*Shigella* is a major cause of severe diarrhea, with *Shigella flexneri* and *Shigella sonnei* accounting for over 90% of infections. Progressive economic growth worldwide correlates with the replacement of *S. flexneri* by *S. sonnei* as the dominant cause of shigellosis. The basis of the epidemiological shift remains unclear, but it highlights the urgent need for further studies on the increasingly prevalent, but less well-studied, *S. sonnei*. Here, we investigated whether *S. sonnei* is better equipped to survive nutrient starvation, a crucial condition for persistence both outside the host and within the colonic lumen. *S. sonnei* exhibited greater survival under long-term nutrient starvation (LTNS) than *S. flexneri*, rapidly activating survival mechanisms. We interrogated the genome of *S. sonnei* using transposon-directed insertion-site sequencing (TraDIS), revealing that metabolic pathways (ATP, nucleotide, and amino acid synthesis) and envelope homeostasis systems (e.g., Tol-Pal complex, Bam complex, peptidoglycan recycling, and RpoE stress response) are conditionally essential for LTNS. TraDIS findings were validated by non-competitive and competitive survival of wild-type and deletion mutant strains. We compared the homology of conditionally essential genes between *S. sonnei* and *S. flexneri* to identify genes potentially involved in differential LTNS survival between the species. However, *S. sonnei* strains in which a single gene was replaced with the *S. flexneri* allele showed wild-type survival phenotypes. This suggests that the divergent survival of these two species may be more complex than a monogenic difference. Together, these data define the molecular adaptations of starvation resistance in *S. sonnei* and provide insights into its epidemiological dominance in high-income countries.

**IMPORTANCE:**

Understanding why *Shigella sonnei* has a higher prevalence than *Shigella flexneri* as a country undergoes economic growth is an important challenge in *Shigella* research. The investigation of their biological and genetic differences is key to tackling the impact of the disease. We discovered that *S. sonnei* resists nutrient deprivation better than *S. flexneri*, suggesting a better adaptation to an extracellular lifestyle and a greater preservation of metabolic capabilities. Using a genome-wide transposon sequencing approach, we uncovered the key pathways behind the survival of *S. sonnei* facing nutrient starvation, which include ATP, nucleotide, and amino acid synthesis as well as maintenance of cell envelope integrity. Comparative analysis between *S. sonnei* and *S. flexneri* did not identify an individual gene responsible for the differing survival and may reflect a multifactorial difference. Our data provide a genome-wide basis for understanding how *S. sonnei* is adapted to nutrient-deprived settings, which may be advantageous in the gut lumen and for environmental survival, potentially contributing to its dominance in high-income countries.

## INTRODUCTION

*Shigella* spp. are gram-negative bacteria of the order Enterobacterales that cause shigellosis or bacillary dysentery. Shigellosis is the second deadliest diarrheal disease, with 30% of fatalities occurring in children below 5 years of age (1). *Shigella* is highly contagious as only 10–100 cells suffice to cause disease (2) and spreads mainly via the fecal-oral route person-to-person (3–6) or by ingesting contaminated water or food (7–10). Fluoroquinolone-resistant *Shigella* is now considered by the World Health Organization as a high-priority pathogen in need of urgent control measures (11). Despite research efforts, no licensed vaccine against shigellosis is currently available (12), although many are in development.

The genus *Shigella* includes four serogroups or species: *Shigella flexneri*, *Shigella sonnei*, *Shigella boydii,* and *Shigella dysenteriae. S. flexneri* and *S. sonnei* cause more than 90% of episodes (13). Although *S. sonnei* infections are typically described as milder (14, 15), comparative studies show that infections caused by both species manifest with similar severity, especially when infecting similar populations (16–18). Interestingly, the geographical distribution of *S. sonnei* and *S. flexneri* varies according to economic development: *S. flexneri* predominates in low-income countries (LICs) and *S. sonnei* in high-income countries (HICs) and middle-income countries (MICs). Importantly, global industrialization and improved sanitation have been accompanied by a comparative rise in *S. sonnei* infections (19–23). However, as *S. flexneri* has been traditionally used in *Shigella* studies, available knowledge on *S. sonnei* is more limited (24). Epidemiological data suggest that hygiene measures that reduce *S. flexneri* infections in HIC are not as efficient at reducing *S. sonnei* (19). Understanding the underlying causes behind this phenomenon is, therefore, crucial. A prominent theory is related to natural immunization: *S. sonnei* has a unique O-antigen that is identical to one serotype (O17) of the environmental bacterium *Plesiomonas shigelloides,* a bacterium that is widely distributed in aquatic environments (25, 26). Exposure to the latter via contact with poorly sanitized waters is common in LICs, and this could provide cross-protection against *S. sonnei* (27). Alternatively, ecological or physiological traits, including differences in interactions with the host or the microbiota, may explain the differing distributions.

*Shigella* is typically described as an intracellular pathogen. However, we have previously shown that the O-antigen of *S. sonnei* reduces Type 3 Secretion System (T3SS) effectiveness, resulting in less macrophage pyroptosis and reduced access to the cytoplasm of intestinal epithelial cells compared to *S. flexneri* (28). Additionally, unlike *S. flexneri*, *S. sonnei* encodes colicins, usually on the plasmid spB, which kill commensal members of the microbiota (29). In a zebrafish infection model, *S. sonnei* causes persistent infections with a high bacterial burden, while only certain *S. flexneri* serotypes persist but at lower bacterial burdens than seen for *S. sonnei* (30). These observations, coupled with their high level of antimicrobial resistance, may give *S. sonnei* a competitive advantage in M/HICs (31).

While most progress in bacteriology has focused on the study of organisms in nutrient abundance, bacteria are often exposed to periods of nutrient scarcity (32). If bacteria cannot acquire nutrients such as nucleobases, amino acids, and vitamins exogenously from the environment, they must produce these nutrients through biosynthetic capabilities. How microbes modulate their physiology when living in starvation is a growing subject of research (33–36). In the context of *Shigella* infections, surviving low nutrient availability has implications both outside and within the host. *Shigella* can be transmitted through fomites and water, and therefore, it must be able to survive nutrient deprivation encountered in these conditions. *S. sonnei* outbreaks have been associated with fountains, lakes, swimming pools, and groundwater, indicating *S. sonnei* can survive in these environments (7, 9, 37–40). Relative to LICs, in HICs, advanced treatment technologies reduce the levels of nutrients in water (41, 42), potentially favoring bacteria better adapted to nutrient-poor conditions. Inside the host, *S. sonnei* exhibits poor invasion of host cells but has acquired mechanisms to compete as an extracellular bacterium with the gut microbiota. In addition to direct competition with the gut microbiota through colicins, *S. sonnei* might have additional strategies to survive colonization resistance. Nutrient starvation is a well-described component of colonization resistance (43), and therefore, the ability to survive nutrient restriction may also benefit *S. sonnei* during gut colonization. We, therefore, investigated the ability of *S. sonnei* to survive nutrient deprivation during long-term nutrient starvation (LTNS) and observed that *S. sonnei* survived LTNS better than *S. flexneri*. We suggest that this may provide *S. sonnei* with a survival advantage both in the environment (in low-nutrient water typical of HIC) and in the colonic lumen (rather than intracellularly within the enterocyte cytosol).

To comprehensively describe the molecular mechanisms used by *S. sonnei* to survive LTNS, we used a genome-wide approach employing transposon-directed insertion site sequencing (TraDIS). TraDIS can be used to assess the genetic requirements for growth or survival under selected conditions such as nutrient scarcity (44–46). The pathways identified in our study as conditionally essential for survival in LTNS include nutrient and energy utilization pathways and envelope homeostasis. We also used these data to attempt to identify why *S. sonnei* survived LTNS better than *S. flexneri*. However, our results suggest that monogenic differences may not explain the distinct LTNS phenotype of these two *Shigella* species.

## RESULTS

### *S. sonnei* is more resistant to nutrient starvation than *S. flexneri*

**Fig 1:**
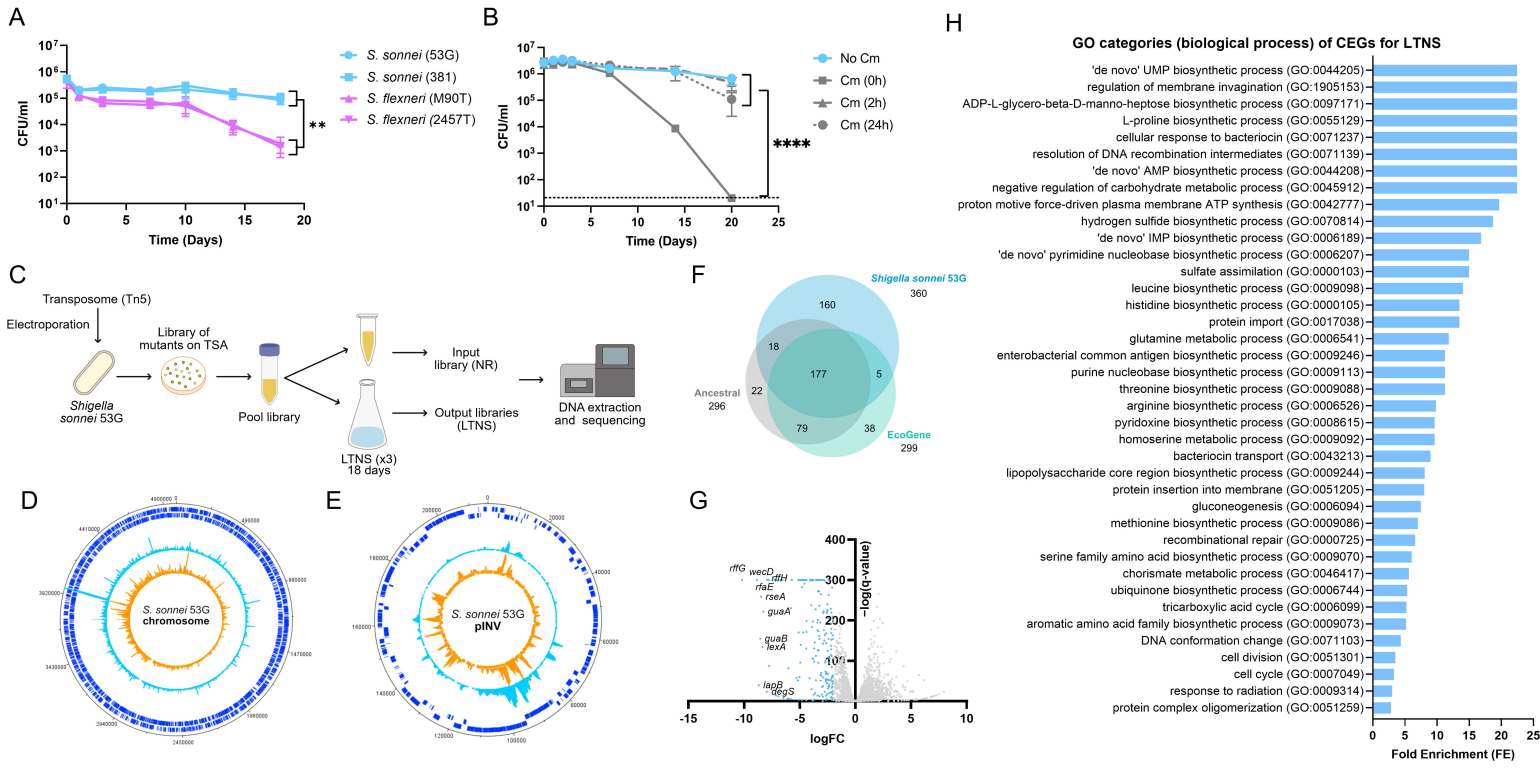
Transposon-directed insertion site sequencing (TraDIS) was used to identify the molecular mechanisms used by *S. sonnei* to survive long-term nutrient starvation (LTNS). (**A**) Survival curves of *Shigella* strains, including *S. sonnei* (53G and 381) and *S. flexneri* (M90T and 2457T), inoculated at an initial concentration of 10^6^ CFU/mL in minimal medium and monitored over 18 days. (**B**) Survival curves of *S. sonnei* 381 in the presence of chloramphenicol added at 0, 2, and 24 h post-inoculation. Survival was compared to that in the absence of Cm and monitored over 20 days. For panels A and B, data show mean values ± SEM of *n* = 3 biological replicates. Log_10_-transformed data were analyzed by two-way ANOVA with the main column effect compared by Tukey’s multiple comparison. **, *P* ≤ 0.01; ****, *P* ≤ 0.0001. (**C**) TraDIS was employed to decipher the genetic mechanisms involved in the survival of *S. sonnei* after LTNS. A schematic representation of the TraDIS workflow is shown. A pool mutant library was generated in nutrient-rich (NR) conditions (input library) and then subjected to LTNS in triplicate (output libraries). The genomic DNA of both input and output libraries was extracted, sequenced, and compared. (**D and E**) Map of transposon insertion sites and frequency in input libraries (NR, orange) and output libraries (LTNS, blue) in *S. sonnei* 53G chromosome (**D**) and the large virulence plasmid, pINV (**E**). (**F**) Venn diagram illustrating the number of essential genes found in *S. sonnei* 53G by TraDIS and their overlap when compared to the essential genes in EcoGene and the ancestral gene set databases. (**G**) Volcano plot of LTNS TraDIS results showing fitness scores for genes in *S. sonnei* 53G chromosome showing their log_2_FC vs adjusted *P*-value represented as −log(*q*-value). Conditionally essential genes (log_2_FC < −2) are highlighted in blue. The 10 genes with the lowest log_2_FC are indicated. (**H**) Significant Gene Ontology categories of conditionally essential genes identified by TraDIS, using the protein analysis through evolutionary relationships (PANTHER) classification system. Only categories with a *P*-value < 0.05 are displayed.

To assess if *S. sonnei* survives LTNS better than *S. flexneri,* we initially analyzed the survival of two strains belonging to each of the two species (M90T and 2457T for *S. flexneri*, 381 and 53G for *S. sonnei*) in M9 media with no carbon source. As can be seen in Fig. 1A, both *S. sonnei* strains maintain CFU numbers above 10^5^ CFU/mL over the long time course. Conversely, *S. flexneri* strains decline abruptly from day 10 to a final concentration of 10^3^ CFU/mL at day 18. This finding led us to investigate how and when *S. sonnei* adapted to nutrient starvation. We supplemented *S. sonnei* M9 cultures with chloramphenicol (Cm), an antibiotic that targets ribosomal function to impede protein production. Chloramphenicol was added at times 0, 2, and 24 h after exposure to M9 media with no carbon source. The number of viable cells was then monitored throughout the course of the experiment (Fig. 1B). Only when Cm was added at 0 h did we observe a reduction in the number of viable cells. Interestingly, the reduction did not occur until after day 7. When Cm was added at 2 or 24 h, *S. sonnei* survival remained unaffected, suggesting that the changes necessary for survival are rapidly activated within the first 2 h of starvation. Collectively, these results support our hypothesis and demonstrate that *S. sonnei* can rapidly adapt to nutrient deprivation to survive better over long time periods compared to *S. flexneri*.

### Transposon-directed insertion-site sequencing reveals gene essentiality and LTNS survival mechanisms of *S. sonnei*

After confirming that *S. sonnei* is more resistant against nutrient depletion than *S. flexneri,* we were intrigued to unveil the molecular mechanisms responsible. For this purpose, we designed a TraDIS approach to compare transposon insertion frequencies across the genome of *S. sonnei* grown in nutrient-rich (NR) conditions (input library) or subjected to LTNS (output library) (Fig. 1C). We used transposon Tn5 to generate a comprehensive mutant library in the representative laboratory strain *S. sonnei* 53G grown in NR conditions. The library consisted of 136,857 independent insertions on the chromosome and 15,430 independent insertions on the large virulence plasmid, pINV (LVP). This indicates an insertion density of approximately 1 insertion per 36 nucleotides on the chromosome and 1 insertion per 14 nucleotides on the LVP. This NR input library was subjected to LTNS, and input/output libraries were sequenced, with insertions mapped to the *S. sonnei* 53G chromosome (Fig. 1D) and the LVP (Fig. 1E).

Genes were classified according to the frequency of insertions. Previous work has established that an insertion density of 1 insertion per 35 nucleotides is the minimum density for accurate prediction of gene essentiality (47). The chromosomal insertion density is on the border of this required density, while the LVP insertion density is well within this requirement. We, therefore, examined essentiality and determined that 419 genes were essential, i.e., there were no insertions in input or output libraries (Table S1). All the predicted essential genes were chromosomally encoded, with the exception of two short predicted pseudogenes (75 and 84 bp, respectively) located on the LVP. Since the LVP is known to be non-essential, these two hits are likely to represent false positives and may not have been covered in the transposon library because of their small size. There were also 35 small genes (encoding proteins of 30 aa or less) on the chromosome that were designated essential but may also be false positives; however, these were retained in our data set for completeness. We mapped our reads to the published *S. sonnei* 53G sequence (HE616528), which identified 59 essential genes within an Enterobacteria phage Mu. We were unable to PCR amplify any region of this phage from our 53G isolate, suggesting our isolate does not contain this phage. We, therefore, reduced the number of essential chromosomally encoded genes to 360 in our *S. sonnei* 53G strain by removing genes encoded in Mu from our data set. For contextualization, we compared our essential gene data set to the genes determined to be essential in Enterobacteriaceae using the EcoGene database (curated for *Escherichia coli* K-12) and the ancestral database (47) (Table S2). EcoGene is a refinement of the Keio collection, a reference for gene essentiality in *Enterobacteriaceae* (48), and the ancestral database reconstructs the ancestral essential genome. We found that 49.17% (177 out of 360) of essential genes in *S. sonnei* 53G overlap with both databases (Fig. 1F; Fig. S1).

To uncover a mechanistic understanding of *S. sonnei* adaptation to nutrient starvation, we focused our study on conditionally essential genes (CEGs) for LTNS, i.e., those with fewer insertions in the output than the input libraries (cutoff = log_2_FC <−2, *q*-value < 0.05) (Fig. 1G). These genes may be required for adaptation to nutrient starvation or continued survival over the long time course. From the chromosome and the LVP, bioinformatic analysis identified 209 CEGs. No conditionally essential or essential genes were identified on the remaining plasmids. The 50 genes with the most significant log_2_FC are listed in Table 1. All CEG genes can be found in Table S3.

**TABLE 1:** Top 50 genes, according to their log_2_FC, identified by TraDIS as conditionally essential for the survival of *S. sonnei* 53G under LTNS

Gene	Function	Log_2_FC	*q*-value
*rffG*	dTDP-glucose 4,6-dehydratase	−10.20	0
*wecD*	dTDP-4-amino-4,6-dideoxy-D-galactose acyltransferase	−8.82	0
*YciM/LapB*	LPS assembly protein B	−8.67	2.76E−40
*guaB*	IMP dehydrogenase	−8.55	9.43E−156
*rseA*	Anti-sigma-E factor RseA	−8.47	2.77E−259
*lexA*	LexA repressor	−8.34	1.05E−134
*guaA*	GMP synthase (glutamine-hydrolyzing)	−8.26	8.14E−223
*degS*	Outer membrane-stress sensor serine endopeptidase DegS	−7.95	4.22E−24
*rfaE*	Bifunctional heptose 7-phosphate kinase/heptose 1-phosphate adenyltransferase	−7.94	0
*rffH*	Glucose-1-phosphate thymidylyltransferase	−7.77	0
*atpE*	ATP synthase subunit C	−7.75	8.49E−88
*waaC*	Lipopolysaccharide heptosyltransferase RfaC	−7.28	0
*lepB*	S26 family signal peptidase	−7.14	9.28E−15
*wecE*	dTDP-4-amino-4,6-dideoxygalactose transaminase	−7.14	0
*waaF*	ADP-heptose-LPS heptosyltransferase 2	−7.03	0
*glmS*	Glutamine-fructose-6-phosphate transaminase (isomerizing)	−6.92	1.79E−12
*rfaD*	ADP-L-glycero-D-mannoheptose-6-epimerase	−6.72	0
*atpD*	ATP synthase subunit beta	−6.59	0
*atpA*	ATP synthase subunit alpha	−6.57	0
*rpiA*	Ribose-5-phosphate isomerase	−6.46	9.19E−09
*secE*	Protein translocase subunit SecE	−6.08	8.35E−07
*atpF*	ATP synthase subunit B	−6.02	4.33E−82
*ribA*	GTP cyclohydrolase II	−5.93	5.62E−06
*wzyE*	O-antigen assembly polymerase	−5.93	5.62E−06
*galU*	UTP-glucose-1-phosphate uridylyltransferase	−5.80	3.92E−231
*pyrG*	CTP synthetase	−5.77	3.69E−05
*surA*	Chaperone SurA	−5.72	0
*higA*	Transcriptional regulator	−5.63	2.02E−19
*tpiA*	Triose-phosphate isomerase	−5.29	1.32E−58
*gmhA*	Phosphoheptose isomerase	−5.16	2.47E−52
*cysQ*	3′(2′),5′-Bisphosphate nucleotidase CysQ	−5.14	0
*rpsF*	30S ribosomal protein S6	−5.10	3.87E−04
*proC*	Pyrroline-5-carboxylate reductase	−5.09	1.04E−170
*prc*	Carboxy terminal-processing peptidase	−5.03	9.48E−61
*purA*	Adenylosuccinate synthetase	−5.02	0
*atpG*	ATP synthase subunit gamma	−4.97	0
*atpC*	ATP synthase epsilon chain	−4.94	2.27E−96
*atpG*	ATP synthase subunit delta	−4.91	5.62E−140
*atpA*	ATP synthase subunit A	−4.88	0
*purK*	5-(Carboxyamino)imidazole ribonucleotide synthase	−4.80	3.29E−283
*folP*	Dihydropteroate synthase	−4.79	2.8E−03
*purH*	Bifunctional phosphoribosylaminoimidazolecarboxamide formyltransferase/inosine monophosphate cyclohydrolase	−4.74	0
*purD*	Phosphoribosylamine-glycine ligase	−4.69	6.30E−171
*wecF*	TDP-N-acetylfucosamine:lipid II N-acetylfucosaminyltransferase	−4.68	0
*carB*	Carbamoyl phosphate synthase large subunit	−4.65	0
*pyrB*	Aspartate carbamoyltransferase catalytic subunit	−4.59	1.16E−291
*acnB*	Aconitate hydratase B	−4.55	0
*recB*	Exodeoxyribonuclease V subunit beta	−4.51	0
*purM*	Phosphoribosylformylglycinamidine cyclo-ligase	−4.43	0
*purC*	Phosphoribosylaminoimidazolesuccinocarboxamide synthase	−4.42	3.43E−131

In addition to the CEGs, there were also 249 conditionally detrimental genes (CDGs); mutants that were enriched during LTNS (cutoff = log_2_FC > 2, *q*-value < 0.05). These included 227 genes on the chromosome, 19 on the LVP, and 1 each on plasmids spB and spC. Ten of the CDGs on the large virulence plasmid were related to the regulation, structure, or function of the T3SS (*mxiDKM, spa40/33/13, ipgA, virF, acp, ospE2*), one was involved in plasmid replication (*tap*), while the remainder were pseudogenes/hypothetical genes. Of the 228 CDGs on the chromosome, many were either unannotated or annotated as hypothetical or pseudogenes. Exceptions of potential further interest included genes encoding cold shock proteins (*cspE* and *G*), Fe-S cluster proteins (*sufC* and *E*), and the RpoS anti-adapters (*iraM* and *D*). However, for the purposes of this study, subsequent analyses focused on the CEGs.

### Analysis of conditionally essential genes and pathways

To uncover the main pathways that allow LTNS survival in *S. sonnei*, we assigned the set of CEGs to GO categories (biological process) (Fig. 1H; Table S4). Thirty-nine significant GO categories (*P*-value < 0.05) were obtained. Pathways with a high fold enrichment include “*de novo”* UMP biosynthetic process (GO:0044205), L-proline biosynthetic process (GO:0055129), proton motive force-driven plasma membrane ATP synthesis (GO:0042777), leucine biosynthetic process (GO:0009098), and histidine biosynthetic process (GO:0000105).

**Fig 2:**
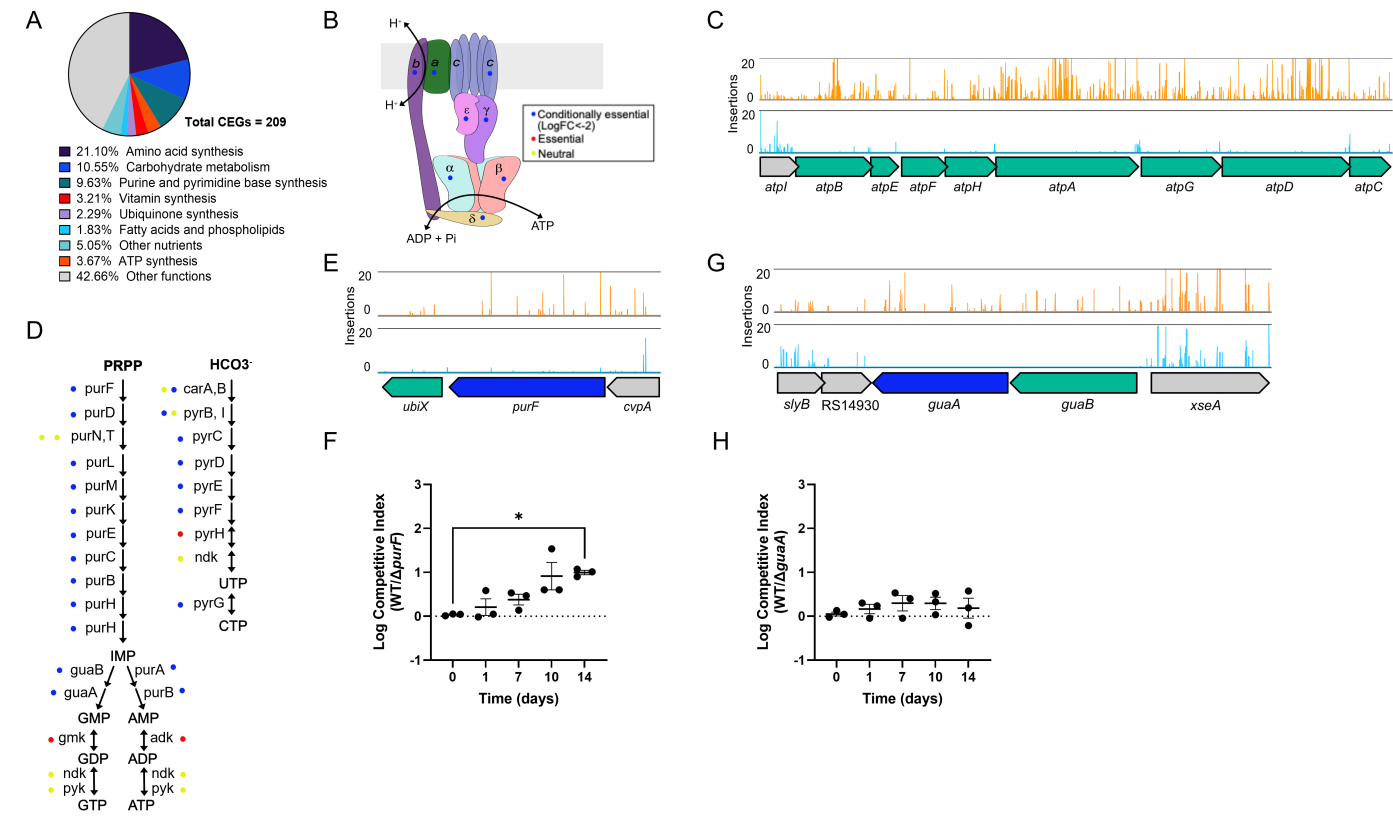
Metabolic pathways, including ATP and nucleotide synthesis pathways, identified by TraDIS as conditionally essential for the survival of *S. sonnei* after LTNS. (**A**) Metabolic genes account for more than half of the conditionally essential genes found in TraDIS analysis. (**B**) ATP synthase subunits. Color dots indicate gene identification by TraDIS as conditionally essemtial (blue), essential (red), or neutral (yellow). (**C**) Transposon insertions in ATP synthesis locus. Read counts are limited to 20. CEGs are colored in green. (**D**) Schematic of purine and pyrimidine synthesis pathways. Color dots indicate gene identification by TraDIS as conditionally essential (blue), essential (red), or neutral (yellow). (**E**) TraDIS insertions in *purF*. Read counts are limited to 20. (**F**) Competition assays between WT SS381 and SS381∆*purF*. Logarithmic values of competitive index are plotted individually and as mean values ± SEM of *n* = 3 biological replicates. The dotted line at log competitive index = 0 represents equal competition fitness. A Kruskal-Wallis with Dunn’s multiple comparisons was used to compare mean values at different time points relative to time 0. *, *P* ≤ 0.05. (**G**) TraDIS insertions in *guaA*. Read counts are limited to 20. *guaA* is colored in blue, and other CEGs are colored in green. (**H**) Competition assays between WT SS381 and SS381∆*guaA*. Logarithmic values of competitive index are plotted individually and as mean values ± SEM of *n* = 3 biological replicates. The dotted line at log competitive index = 0 represents equal competition fitness. A one-way ANOVA with Dunnett’s multiple comparisons was used to compare mean values at different time points relative to time 0. No significant differences were found.

Our results reveal that among the CEGs *S. sonnei* requires to survive LTNS, more than half (*n* = 125/209) participate in the metabolism of major nutrients and energy: carbohydrates, amino acids, nucleotides, nitrogenous bases, vitamins, and ATP (Fig. 2A). These findings not only align with expectations and reinforce the validity of our experimental approach, but they also reveal the critical metabolic requirements for *S. sonnei* facing nutrient deprivation. Genes encoding enzymes that participate in the central metabolism of carbohydrates were found to be conditionally essential: these include key glycolysis enzymes such as *pgm* (phosphoglucomutase) and *pgi* (phosphoglucose isomerase), along with several enzymes of the tricarboxylic acid cycle, including *acnB* (aconitase B), *gltA* (citrate synthase), and *sucA* and *sucB* (components of the 2-oxoglutarate dehydrogenase complex). Glycolysis produces pyruvate that can be incorporated into the tricarboxylic acid cycle to produce energy and precursors for the synthesis of various biomolecules. Additionally, genes involved in the synthesis of glycogen from glucose (*glpD, glgA,* and *glgC*) and gluconeogenesis genes (*tpiA, pgi,* and *fbp*) were found among this set of genes, suggesting that the production of glycogen from non-carbohydrate sources is also vital under starvation conditions. The synthesis of at least 13 amino acids (methionine, threonine, leucine, isoleucine, valine, proline, tryptophan, histidine, cysteine, tyrosine, arginine, glutamine, and serine) was found to be conditionally essential. Likewise, the majority of genes involved in the biosynthesis of nitrogenous bases, both purines and pyrimidines, together with the locus that encodes for the ATP synthase complex, were found among this set. Genes encoding nucleotide and amino acid synthesis functions were selected for individual testing to validate TraDIS findings.

### Metabolic pathways of purine and amino acid synthesis are crucial for the survival of *S. sonnei* in LTNS

We constructed deletion mutants of selected CEGs in the *S. sonnei* strain SS381 and assessed the effect of these mutations by comparing survival in single-strain cultures, as well as in competition assays with the wild-type (WT) strain. We chose to use SS381, given that this strain demonstrates the same survival as the 53G strain during LTNS (Fig. 1A) and is representative of a more epidemiologically prevalent lineage of *S. sonnei* (i.e., Lineage 3). To discriminate between WT and mutant strains during competition assays, we introduced a gene encoding green fluorescent protein (GFP) downstream of *glmS* (glucosamine-fructose-6-phosphate aminotransferase) in SS381, a site of insertion previously identified as neutral (49). We ensured that this *gfp* insertion had no effect on SS381 survival during LTNS and could therefore be used as the WT (Fig. S2). For comparison, the growth of all strains used in this study in NR conditions can be found in Fig. S3.

Since most genes encoding for the ATP synthase complex were identified as conditionally essential (Fig. 2B and C), and this complex has a central role in energy conservation, we initially targeted genes *atpA* and *atpE* for mutagenesis. Despite numerous attempts, we were unable to obtain deletions of these genes. Purine and pyrimidine synthesis pathways also contained many CEGs (Fig. 2D). Therefore, we decided to target genes in these pathways to confirm the TraDIS results. PurF (amidophosphoribosyltransferase) initiates the purine biosynthesis pathway by converting 5-phosphoribosyl-α-1-pyrophosphate to 5-phosphoribosylamine, and we were able to successfully construct a deletion mutant of this gene. This pathway produces inosine monophosphate, the precursor of adenine and guanine nucleotides (Fig. 2D and E). Δ*purF* survival was assessed in single-strain cultures and in our competition assay. In single-strain cultures, the difference in survival between WT and Δ*purF* was not significant (Fig. S4A), while in the competition assay, Δ*purF* demonstrated significant survival defects compared to the WT strain (Fig. 2F). We also constructed a mutant in *guaA*, a guanosine monophosphate (GMP) synthetase, which catalyzes the last step of GMP synthesis (Fig. 2D and G). In single-strain cultures, Δ*guaA* did not show any impairment in comparison to the WT, while in the competition assay, the WT showed an advantage, albeit without reaching statistical significance (Fig. S4B; Fig. 2H). This suggests that *de novo* nucleotide synthesis is important for LTNS survival.

**Fig 3:**
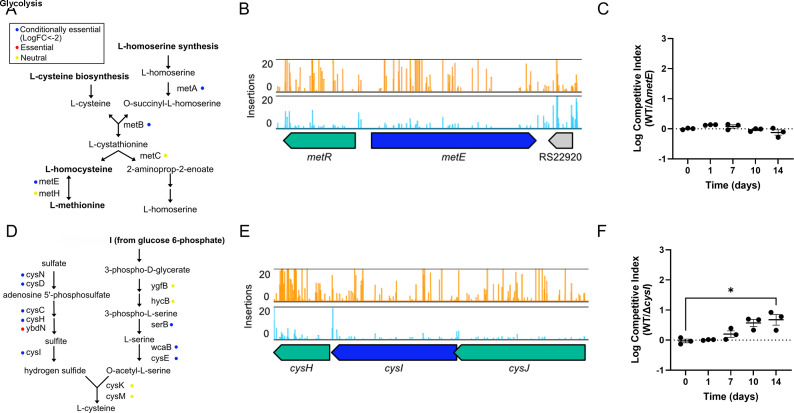
Multiple amino acid synthesis pathways are conditionally essential LTNS. (**A**) Schematic of the methionine synthesis pathway. Color dots indicate gene identification by TraDIS as conditionally essential (blue), essential (red), or neutral (yellow) for all figure panels. (**B**) Transposon insertions in *metE*. Read counts are limited to 20. *metE* is colored in blue, and other CEGs are colored in green. (**C**) Competition assays between WT SS381 and SS381∆*metE*. Logarithmic values of competitive index are plotted individually and as mean values ± SEM of *n* = 3 biological replicates. The dotted line at log competitive index = 0 represents equal competition fitness. A one-way ANOVA with Dunnett’s multiple comparisons was used to compare mean values at different time points relative to time 0. No significant differences were found. (**D**) Cysteine synthesis pathway. (**E**) Transposon insertions in *cysI*. Read counts are limited to 20. *cysI* is colored in blue, and other CEGs are colored in green. (**F**) Competition assays between WT SS381 and SS381∆*cysI*. Logarithmic values of competitive index are plotted individually and as mean values ± SEM of *n* = 3 biological replicates. The dotted line at log competitive index = 0 represents equal competition fitness. A one-way ANOVA with Dunnett’s multiple comparisons was used to compare mean values at different time points relative to time 0. *, *P* ≤ 0.05.

We next constructed deletion mutants in selected amino acid synthesis pathways: methionine (∆*metE*) and cysteine (∆*cysI*). These mutants were tested in our single-strain survival and competition assays to determine their conditional essentiality during LTNS. MetE, a cobalamin-independent homocysteine transmethylase, catalyzes the final step of methionine biosynthesis (Fig. 3A and B). ∆*metE* showed no impairment in the single-strain culture (Fig. S4C). In competition with the WT, early time points suggested a slight advantage of the WT (day 1), but survival levels were comparable at later time points. This may be due to MetH, the cobalamin-dependent homocysteine transmethylase compensating for the MetE deletion (Fig. 3A and C). CysI and CysJ are subunits of the sulfate reductase that produces sulfate necessary for cysteine synthesis (Fig. 3E and F). A deletion in *cysI* again showed no impairment in the single-strain culture (Fig. S4D) but did have significantly reduced survival compared to WT during the competition assay (Fig. 3F).

Together, these results validate our TraDIS findings, highlighting nucleotide and amino acid synthesis pathways as important for *S. sonnei* to survive LTNS. These pathways are crucial for maintaining the nucleotide pools required for DNA and RNA synthesis and repair, and the amino acid pool for protein synthesis under nutrient-restricted conditions. Furthermore, we demonstrate the sensitivity of our competition assay in determining survival differences between strains.

### Cell envelope homeostasis has a role in the survival of *S. sonnei* in LTNS

Most CEGs identified in this study belong to nutrient and metabolic pathways; however, categories related to envelope homeostasis were also highlighted (Fig. 1H). Genes involved in the synthesis and regulation of molecules present in the cell envelope were also substantially represented in categories such as regulation of membrane invagination (GO:1905153), enterobacterial common antigen biosynthetic process (GO:0009246), lipopolysaccharide core region biosynthetic process (GO:0009244), and protein insertion into membrane (GO:0051205). To corroborate these findings, we investigated the effect of mutations in pathways participating in the homeostasis of envelope components, including phospholipids, outer membrane proteins (OMPs), and peptidoglycan (PG).

**Fig 4:**
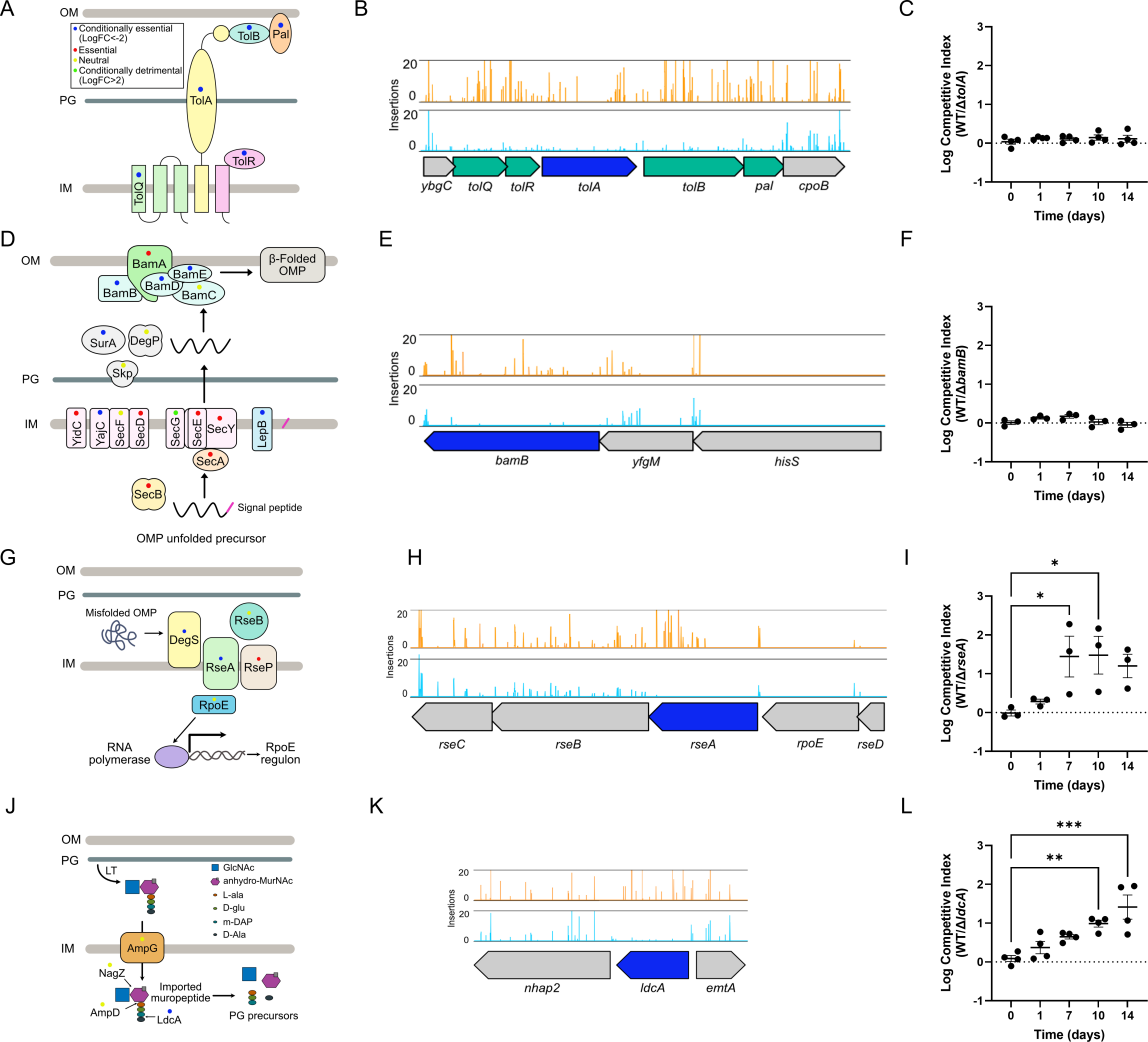
Maintaining cell envelope homeostasis contributes to LTNS survival. (**A**) Schematic representation of the Tol-Pal system in the cell envelope. Color dots indicate gene identification by TraDIS in all figure panels as conditionally essential (blue), essential (red), neutral (yellow), or conditionally detrimental (green). (**B**) Transposon insertions in genes encoding the Tol-Pal complex. Read counts are limited to 20. *tolA* is colored in blue, and other CEGs are colored in green. (**C**) Competition assays between WT SS381 and SS381∆*tolA*. Logarithmic values of competitive index are plotted individually and as mean values ± SEM of at least three biological replicates. The dotted line at log competitive index = 0 represents equal competition fitness. A one-way ANOVA with Dunnett’s multiple comparisons was used to compare mean values at different time points relative to time 0. No significant differences were found. (**D**) Schematic representation of the Bam complex in the cell envelope. (**E**) Transposon insertions in *bamB*. Read counts are limited to 20. *bamB* is colored in blue. (**F**) Competition assays between WT SS381 and SS381∆*bamB*. Logarithmic values of competitive index are plotted individually and as mean values ± SEM of *n* = 3 biological replicates. The dotted line at log competitive index = 0 represents equal competition fitness. A one-way ANOVA with Dunnett’s multiple comparisons was used to compare mean values at different time points relative to time 0. No significant differences were found. (**G**) Schematic representation of the σ^E^ extracytoplasmic stress response. (**H**) Transposon insertions in *rseA*. Read counts are limited to 20. *rseA* is colored in blue. (**I**) Competition assays between WT SS381 and SS381∆*rseA*. Logarithmic values of the competitive index are plotted individually and as mean values ± SEM of *n* = 3 biological replicates. The dotted line at log competitive index = 0 represents equal competition fitness. A one-way ANOVA with Dunnett’s multiple comparisons was used to compare mean values at different time points relative to time 0. *, *P* ≤ 0.05. (**J**) Schematic representation showing the role of *ldcA* acting on the imported muropeptide during PG recycling. (**K**) Transposon insertions in *ldcA*. Read counts are limited to 20. *ldcA* is colored in blue. (**L**) Competition assays between WT SS381 and SS381∆*ldcA*. Logarithmic values of the competitive index are plotted individually and as mean values ± SEM of at least three biological replicates. The dotted line at log competitive index = 0 represents equal competition fitness. A one-way ANOVA with Dunnett’s multiple comparisons was used to compare mean values at different time points relative to time 0. **, *P* ≤ 0.01; ***, *P* ≤ 0.001.

The five proteins encoding the Tol-Pal transmembrane complex were conditionally essential for survival in LTNS (Fig. 4A and B). This system participates in membrane invagination, removes excessive phospholipids from the outer membrane (OM), and ensures envelope stability by linking the OM, PG, and inner membrane (IM) (50–53). TolA extends across the periplasm, connecting the OM and IM components of the system (Fig. 4A). We constructed a deletion mutant of *tolA,* which survived in single-strain cultures at a level equivalent to WT (Fig. S4E). When in competition with WT, Δ*tolA* had a slight reduction in survival from day 1 of LTNS, although this was not statistically significant (Fig. 4C).

OMPs are generated in the cytoplasm and transported to the OM (Fig. 4D). Initially, unfolded OMPs cross the IM with the aid of the Sec system directed by their amino-terminal signal peptide. The Sec system is ubiquitous in bacteria and is formed by the SecYEG translocon, auxiliary proteins SecDF-YajC-YidC, and chaperones SecAB (54). Following cleavage of the signal peptide by LepB, OMPs are transported to the OM by periplasmic chaperones (SurA, Skp, DegP, and FkpA). OMPs are then folded in their β-barrel conformation and inserted into the OM by the β-barrel assembly machinery complex, made up of five proteins; BamA anchored in the OM and lipoproteins BamB, BamC, BamD, and BamE (55). Our analysis found the majority of genes in these pathways to be either conditionally essential (SecE, YajC, LepB, SurA, BamB, and BamD) or essential for viability (i.e., no insertions found in any study condition, SecY, SecB, YidC, and BamA). We selected the CEG *bamB* for deletion and characterization under LTNS (Fig. 4E). Δ*bamB* survived in single-strain cultures at a level equivalent to WT (Fig. S4F), while in the competition assay, Δ*bamB* had a slight disadvantage at early time points (day 1 and day 7), while at the later time points, its recovery was equivalent to WT (Fig. 4F).

RseA is the anti-sigma factor of σ^E^, a key regulator of the extracytoplasmic stress response and one of the sensory pathways (together with Cpx, Rcs, and Psp) that respond to envelope stress (56). The σ^E^ response activates upon the presence of unfolded OMPs in the periplasm or altered LPS forms. This causes an enzymatic cascade leading to RseA disassociation from σ^E^, which in turn associates with the RNA polymerase for the expression of target genes (57) (Fig. 4G). *rseA* ranked as the fifth most impaired gene in our screening (Fig. 4H), so it was selected for mutagenesis. In single-strain cultures, there was a significant difference in survival between WT and ∆*rseA* from day 7 post-starvation (Fig. S4G). In the competition assay, ∆*rseA* had significant survival defects compared to the WT strain (Fig. 4I). This highlights the importance of envelope stress responses in sustaining LTNS survival.

Most genes involved in PG synthesis are essential for survival; however, *ldcA* was found to be conditionally essential. LdcA is a carboxypeptidase that participates in PG recycling by releasing the terminal D-alanine residue from the cytoplasmic tetrapeptide, allowing reuse of the tripeptide by the former (58) (Fig. 4J and K). ∆*ldcA* had significantly reduced survival in LTNS compared to WT in the single-strain culture from day 7 (Fig. S4H). In competition assays, WT showed a strong advantage in comparison to ∆*ldcA*, indicating a requirement for survival in LTNS (Fig. 4L). This finding infers that PG recycling is key to surviving starvation, potentially due to a reduction in *de novo* PG synthesis.

### Identifying genes responsible for the difference between *S. sonnei* and *S. flexneri* survival of LTNS

**Fig 5:**
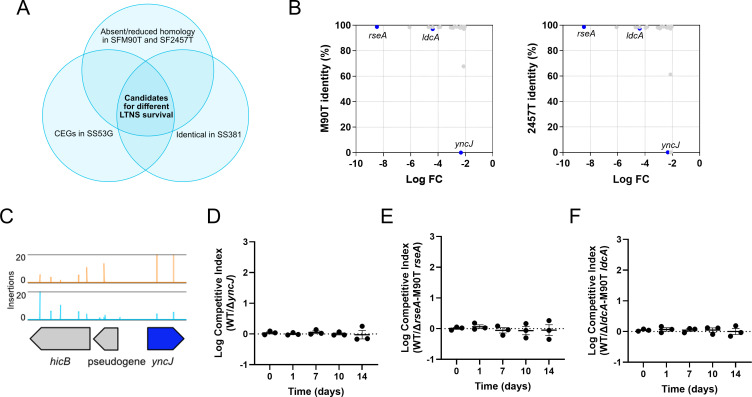
Identification of potential genetic markers that explain the different ability of *S. sonnei* and *S. flexneri* to resist LTNS. (**A**) Venn diagram showing the chosen criteria for the identification of genetic candidates potentially explaining the different ability of *S. sonnei* and *S. flexneri* to resist LTNS: being conditionally essential in *S. sonnei* 53G, showing absent or reduced homology in *S. flexneri* M90T and *S. flexneri* 2457T, and being identical in *S. sonnei* 381. (**B**) Twenty-four genetic candidates sharing <99% identity with both *S. flexneri* M90T and *S. flexneri* 2457T according to their log FC and amino acid identity. (**C**) Transposon insertions in *yncJ*. Read counts are limited to 20. *yncJ* is colored in blue. (**D**) Competition assays between WT SS381 and SS381∆*yncJ*. Logarithmic values of competitive index are plotted individually and as mean values ± SEM of *n* = 3 biological replicates. The dotted line at log competitive index = 0 represents equal competition fitness. A Kruskal-Wallis with Dunn’s multiple comparisons was used to compare mean values at different time points relative to time 0. No significant differences were found. (**E**) Competition assays between WT SS381 and SS381∆*rseA-*M90T*rseA*. Logarithmic values of competitive index are plotted as mean values ± SEM of *n* = 3 biological replicates. The dotted line at log competitive index = 0 represents equal competition fitness. A one-way ANOVA with Dunnett’s multiple comparisons was used to compare mean values at different time points relative to time 0. No significant differences were found. (**F**) Competition assays between WT SS381 and SS381∆*ldcA-*M90T*ldcA*. Logarithmic values of competitive index are plotted as mean values ± SEM of *n* = 3 biological replicates. The dotted line at log competitive index = 0 represents equal competition fitness. A one-way ANOVA with Dunnett’s multiple comparisons was used to compare mean values at different time points relative to time 0. No significant differences were found.

To investigate genetic candidates that could explain the difference in LTNS survival between *S. flexneri* and *S. sonnei,* we compared the protein sequence of each CEG identified in *S. sonnei* 53G with their counterpart in *S. flexneri* M90T and 2457T. Genes of interest were those absent or non-homologous in the *S. flexneri* genomes (Table S5). We further ensured that the putative candidates were conserved in *S. sonnei* 381 (Fig. 5A and B). This analysis identified one promising candidate that was absent from both *S. flexneri* genomes and 100% identical in the *S. sonnei* genomes: *yncJ* (Fig. 5C). YncJ is a largely uncharacterized protein that contains a domain of unknown function (DUF2554) and appears to be restricted to *Enterobacteriaceae*. We constructed a *∆yncJ* strain and analyzed survival in our LTNS assays. However, this mutant did not show a significantly decreased survival in LTNS compared to WT in either single-strain cultures or in competition assays (Fig. S4I; Fig. 5D). Therefore, *yncJ* is not a genetic marker that explains the difference in LTNS survival between *S. sonnei* and *S. flexneri*.

We then broadened our analysis, focusing on genes that were non-homologous between *S. sonnei* and *S. flexneri* genomes. One hundred and nine CEGs met our criteria (100% amino acid identity in *S. sonnei* genomes and <100% amino acid identity in both *S. flexneri* genomes). In order to choose candidates that could be responsible for the difference between *S. sonnei* and *S. flexneri,* we combined information on the homology between the species (looking for non-conservative aa changes) and the log_2_FC from TraDIS. We identified 24 candidates that share <99% identity with both M90T and 2457T (Table 2; Fig. 5B). Among these, we focused on *ldcA* and *rseA*, both shown here to be required for LTNS survival in *S. sonnei* (Fig. S4G; Fig. 4I; Fig. S4H; Fig. 4L). We next tested whether allelic differences in these genes could explain the differential survival between *S. sonnei* and *S. flexneri*.

**TABLE 2:** CEGs identified in *S. sonnei* 53G that show less than 99% identity with both *S. flexneri* M90T and 2457T

Gene	Log_2_FC	M90T identity (%)	2457T identity (%)
*yncJ*	−2.33	0	0
*ybcO*	−2.13	67.71	61.22
*ldcA*	−4.39	97.04	97.36
*frsA*	−2.10	97.06	0
*yejL*	−2.12	97.33	97.33
*hisIE*	−2.90	98.03	97.54
*lnt*	−2.94	98.05	97.66
*rfaP*	−2.40	98.11	98.11
*rfaH*	−2.31	98.15	98.15
*ruvC*	−3.07	98.27	98.27
*pyrF*	−3.96	98.37	97.96
*secE*	−6.08	98.43	98.43
*ubiH*	−2.25	98.47	98.47
*pyrE*	−4.40	98.59	98.59
*purD*	−4.69	98.6	98.37
*glpD*	−3.88	98.6	98.76
*ilvY*	−2.83	98.65	98.65
*rseA*	−8.47	98.71	98.61
*hemC*	−3.84	98.72	98.72
*cysN*	−2.18	98.74	98.95
*pdxJ*	−2.35	98.77	98.77
*moeB*	−2.05	98.8	98.8
*cysJ*	−2.63	98.83	98.83
*infB*	−2.04	98.88	98.99

*rseA* had the highest log_2_FC of our candidates (Table 2), and there are three amino acid differences between *S. sonnei* and *S. flexneri* RseA, one of which is non-conservative and within the NT σ^E^ binding region (Fig. S5A) (59). We complemented ∆*rseA* with the M90T *rseA* and compared this to WT in non-competitive conditions and in a competition assay. Unexpectedly, the M90T *rseA* fully complemented ∆*rseA* with no significant difference at any time point between the WT and ∆*rseA-*M90T*rseA* in both experiments (Fig. S4J; Fig. 5E). Therefore, differences between *S. flexneri* and *S. sonnei* alleles of *rseA* alone do not explain the difference in LTNS survival between these species.

*ldcA* has a high log_2_FC (Table 2), and there are nine amino acid differences between *S. sonnei* and *S. flexneri* LdcA, three of which are non-conservative (Fig. S5B). Complementation with M90T *ldcA* showed no significant differences between the WT and the complemented strains (Fig. 5E and F), indicating different *ldcA* alleles are not responsible for the differential survival. These findings suggest that a single gene may not be responsible for the difference in LTNS survival between *S. flexneri* and *S. sonnei,* but rather multiple genetic or transcriptional differences may be involved.

## DISCUSSION

The epidemiological landscape of shigellosis is evolving, with *S. sonnei* progressively replacing *S. flexneri* in countries undergoing economic growth. Therefore, understanding the differences between these two species is crucial to mitigating the impact of the disease. Here, we show that *S. sonnei* is more resistant to LTNS than *S. flexneri,* potentially contributing to the dominance of *S. sonnei* in HIC. Environmental transmission of *S. sonnei* is thought to be lower than that of *S. flexneri* (60), and while this seems plausible in LIC, evidence suggests an increase in potentially environmentally transmitted *S. sonnei* infections in HIC (61). Since improved water sanitation reduces fecal contamination and nutrient levels in water, the ability of *S. sonnei* to survive LTNS could provide an advantage compared to *S. flexneri* in highly sanitized water used in HIC. In addition, nutrient restriction in the lumen of the colon by resident microbiota will also create a nutrient-deprived environment, which may facilitate *S. sonnei* infection.

*Shigella* spp. are well documented to have undergone genome degradation, reducing the metabolic capabilities compared to the ancestral *E. coli*. Among *Shigella* spp., metabolic modeling studies indicate that *S. sonnei* has retained more metabolic capabilities than *S. flexneri* (62, 63), which is consistent with our results. The enhanced LTNS resistance of *S. sonnei* may allow it to survive extended periods extracellularly within the colonic lumen or within the environment. Surviving nutrient limitation could compensate for the reduced intracellular invasion levels of *S. sonnei* (mediated by the O-Ag in the LPS and the capsule) (28) by reducing the dependence on cell invasion for the acquisition of nutrients. The commensal microbiota restricts pathogen colonization through nutrient limitation (64), and the ability to survive LTNS, together with the expression of colicins by most *S. sonnei* clinical isolates (29), could be advantageous for *S. sonnei* to survive in the gut lumen. Surviving starvation can also result in persistent infections (65). Therefore, our findings may help to explain why *S. sonnei* causes more persistent infection than *S. flexneri* in the zebrafish infection model (30).

We applied a genome-wide TraDIS approach to uncover the genetic mechanisms important for LTNS survival in *S. sonnei*. We identified 209 genes as conditionally essential, and more than 50% of those participate in metabolic pathways such as the synthesis of ATP, nucleotides, and amino acids. Interestingly, many of the pathways identified previously as important for enterohemorrhagic *E. coli* (EHEC) colonization of the gastrointestinal tract were also identified in our study: purine, pyrimidine, methionine, threonine, leucine, isoleucine, valine, proline, tryptophan, histidine, tyrosine, arginine, glutamine, and serine synthesis (66, 67). These convergences highlight the significant overlap between pathways required for surviving nutrient starvation and those required for surviving gastrointestinal colonization, supporting again our hypothesis that *S. sonnei* may retain metabolic pathways that support survival in the gastrointestinal tract.

Our data also highlighted the importance of envelope homeostasis in sustaining LTNS survival. All components of the Tol-Pal system were conditionally essential, despite a single deletion of *tolA* not causing a large impairment in LTNS survival. Previous studies demonstrated that the Tol-Pal system is important for surviving or recovering from nutrient limitation (50). The Tol-Pal system links the OM, PG, and IM (50) and mediates OM invagination, supporting proper envelope remodeling (51). Furthermore, Tol-Pal has been implicated in retrograde phospholipid transport from the OM to the IM (52). The importance of phospholipid transport is also highlighted by the identification of *mlaE*, part of the MlaFEDB phospholipid transport complex, as a CEG in our TraDIS. Interestingly, components of the Tol-Pal complex were also identified as important for gut colonization in EHEC (67). Additionally, our analysis found genes that participate in the biogenesis of OMPs (e.g., Bam complex, Sec complex, and periplasmic chaperones) as CEGs. We deleted *bamB,* although again this single deletion did not cause significant defects in LTNS survival. Deletion of *bamB* in *E. coli* causes defects in envelope integrity, likely through interactions with peptidoglycan synthesis (53), which may explain its identification as a CEG. Mutations in *bamB* also show reduced gut colonization in *K. pneumoniae* (68), highlighting again the convergence of genes and pathways required for LTNS survival and enterobacterial gut colonization, demonstrating the value of our workflow in underscoring biologically relevant pathways for *S. sonnei* pathogenesis. RseA, the anti-sigma factor of the rpoE extracytoplasmic stress response, was also found among the CEGs, and we demonstrate that its deletion leads to a severe impairment under LTNS both in single culture and in competition assays. In the absence of *rseA*, the RpoE regulon is uncontrolled, resulting in constitutive expression that leads to envelope defects such as defective OM permeability and decreased OMP levels (57). Additionally, deletion of *rseA* has been associated with cell lysis in prolonged stationary phase (69). We also found LdcA, a carboxypeptidase that participates in PG recycling, to be conditionally essential for LTNS. Most genes that participate in the synthesis of PG are essential due to the crucial role of PG in cell strength and integrity (58). We found that a deletion of *ldcA* leads to impaired survival in LTNS both in single culture and in competition, emphasizing the role of PG recycling when *de novo* synthesis is likely to be minimal. Together, our results support a model in which envelope homeostasis is central for *S. sonnei* survival in starvation, likely by ensuring normal integrity/permeability that may prevent cell lysis and loss of internal components.

We and others have observed discrepancies between results derived from TraDIS and results using deletion mutants (46, 70, 71), reinforcing the importance of validating genome-wide analysis with individual testing. For instance, despite being identified as conditionally essential by TraDIS, *metE* showed slightly decreased fitness at D1 but slightly increased fitness at D14 relative to the WT. This may be due to our sampling technique, where all time points were harvested from the same culture, which can produce discordant results compared to harvesting each time point from a separate culture (72). It is important to remember that TraDIS compares the fitness of each mutant within a highly mixed population and between two conditions. Our single-strain and competition assays are only partially recapitulating this complex comparison. Another important consideration is the specific mutation created. Transposon insertion can lead to partial gene disruption or polarity effects on downstream genes, which may differ from the complete loss of function derived from deletion (45, 73). However, TraDIS remains a valuable approach for investigating bacterial phenotypes (44), and we found that the majority of deletion mutants recapitulated the TraDIS results. Notably, we also found competition assays to be more sensitive to survival differences than single-strain cultures in LTNS. Bacterial fitness has generally been quantified by measuring growth in monoculture under a single stress, but competition assays are more aligned with the evolutionary biology idea of fitness as they measure comparative fitness rather than absolute fitness (74). For enteric bacteria, competition assays in a nutrient-restricted environment are an accurate reflection of their natural habitat (75).

Our study identified 360 essential genes (required under both NR and LTNS conditions) in *S. sonnei* 53G, compared to the previously reported 498 essential genes in *S. sonnei* (76). The previous study used a different strain of *S. sonnei* (ATCC 29930) and achieved a lower number of unique insertion sites than in our current study, which may contribute to the different numbers of essential genes discovered. We anticipate that the actual number of essential genes in *S. sonnei* 53G will be lower than 360, as evidenced by the 2 pseudogenes identified as essential on the non-essential LVP, and the similar small size of at least 35 “essential” genes on the chromosome that may be false positives. A denser library is required to accurately identify the complement of essential genes for *S. sonnei*. False negatives can also occur, especially where insertions have occurred in non-essential regions of essential genes. *glmS* is a well-documented example where C-terminal insertions are permissive, although the entire gene is essential, and indeed, *glmS* was identified as non-essential in our analysis. Consistent with previous evidence, we were unable to make a *glmS* deletion mutant, supporting the essentiality of this gene in *S. sonnei*.

Interestingly, genes that were previously identified as uniquely essential for *S. flexneri* were not essential in our analysis of *S. sonnei* (77). Many of these are hypothesized to have become essential in *S. flexneri* due to the loss of related metabolic pathways, and their non-essentiality *in S. sonnei* reflects that this bacterium has retained more metabolic capabilities of the *E. coli* ancestor (62, 63).

We interrogated our CEGs to determine if a single gene could explain the difference in LTNS survival between *S. sonnei* and *S. flexneri*. While *yncJ* appeared to be a promising candidate, deletion of this gene did not affect *S. sonnei* survival of LTNS. Only two unique insertion sites were found in this gene due to its small size (228 nt). Medium-density libraries such as ours can lead to overestimation of essential and conditionally essential genes (78). For this reason, a second gene, which we identified as a candidate for the differential survival of *S. sonnei* and *S. flexneri, ybcO,* was not investigated further as it was a similar size (291 nt) and again contained only two unique insertion sites. We focused instead on genes that had higher insertion frequencies combined with non-homology between *S. flexneri* and *S. sonnei*. The two genes we tested, *ldcA* (a PG carboxypeptidase) and *rseA* (the anti-sigma factor regulator), have roles in envelope homeostasis and were confirmed to be important for *S. sonnei* survival during LTNS. However, complementation with the *S. flexneri* allele restored the survival to WT levels, indicating that individually these genes were not responsible for the differential survival of *S. flexneri* and *S. sonnei* to LTNS. It is, therefore, likely that a combination of factors contributes to the differential survival in LTNS of these two *Shigella* species rather than a single gene difference.

Taken together, here we have demonstrated significant differences in the abilities of *S. flexneri* and *S. sonnei* to survive nutrient starvation. This finding provides new evidence underscoring the divergence between these two *Shigella* species that may contribute to explaining their different epidemiology and pathogenesis.

## MATERIALS AND METHODS

### Bacterial strains and growth conditions

Bacterial strains used in this study are listed in Table 3. *Shigella* strains were routinely grown in tryptic soy broth (TSB) or tryptic soy agar (TSA) plates supplemented with 0.01% Congo Red, unless otherwise stated. Congo Red allows identification of colonies that harbor the large virulence plasmid (79, 80). *E. coli* strains were grown in lysogeny broth or lysogeny agar plates. Cultures were incubated overnight at 37°C. If required, antibiotics were added to the media with the following concentrations: chloramphenicol (30 µg/mL), gentamicin (20 µg/mL), and kanamycin (Kn; 50 μg/mL).

**TABLE 3:** Bacterial strains used in this study

Strain name	Description	Origin
*S. sonnei* H140860381 (SS381)	Clinical isolate, lineage 3	(28)
*S. sonnei* 53G	Lab-adapted isolate, lineage 2	(81)
*S. flexneri* M90T	Serotype 5a	(82)
*S. flexneri* 2457T	Serotype 2a	(80)
*S. sonnei* LVP^Cm^	*S. sonnei* 53G with *cat* (conferring chloramphenicol resistance) inserted at nt 83,716 of 53G LVP, allowing selection of LVP-positive strains	(28)
SS381 (GFP)	Expresses GFP	This study
SS381 Δ*purF*	SS381 with *purF* deleted by homologous recombination	This study
SS381 Δ*guaA*	SS381 with *guaA* deleted by homologous recombination	This study
SS381 Δ*metE*	SS381 with *metE* deleted by homologous recombination	This study
SS381 Δ*cysI*	SS381 with *cysI* deleted by homologous recombination	This study
SS381 Δ*tolA*	SS381 with *tolA* deleted by homologous recombination	This study
SS381 Δ*bamB*	SS381 with *bamB* deleted by homologous recombination	This study
SS381 Δ*rseA*	SS381 with *rseA* deleted by homologous recombination	This study
SS381 Δ*ldcA*	SS381 with *ldcA* deleted by homologous recombination	This study
SS381 Δ*yncJ*	SS381 with *yncJ* deleted by homologous recombination	This study
SS381 Δ*rseA*-M90T*rseA*	SS381 with *rseA* deleted by homologous recombination and complemented with *rseA* allele from *S. flexneri* M90T	This study
SS381 Δ*ldcA*-M90T*ldcA*	SS381 with *ldcA* deleted by homologous recombination and complemented with *ldcA* allele from *S. flexneri* M90T	This study
*E. coli* CC118λpir	Strain to maintain pSEVA-612S	(83)
*E. coli* 1047 pRK2013	Helper strain in triparental conjugation to transfer pSEVA-612S from CC118λpir to *S. sonnei*	(84)

### Long-term nutrient starvation cultures

To compare the survival of *S. sonnei* and *S. flexneri* strains in long-term nutrient starvation conditions, we used minimal media M9 (33.7 mM Na_₂_HPO_₄_, 22 mM KH_₂_PO_₄_, 8.55 mM NaCl, and 9.35 mM NH₄Cl) supplemented with 2 mM MgSO_₄_ and 0.1 mM CaCl. Strains were first grown in TSB to the exponential phase, centrifuged, and washed with PBS prior to inoculation in M9. Independent M9 cultures of each strain at a concentration of 10^6^ cells/mL were incubated at 23°C ± 2°C, and the number of CFUs was monitored over a period of 18 days. At specific time points, aliquots were collected, and serial dilutions were plated onto TSA to quantify viable CFUs.

To test for differences in the survival in LTNS between different strains derived from *S. sonnei* SS381, we additionally carried out competition experiments. Strains were first grown independently in TSB to the exponential phase, washed with PBS, and inoculated together in M9 at a concentration of 10^6^ cells/mL per strain. Cultures were incubated at 23°C ± 2°C, aliquots were taken over 14 days at different time points, and CFUs were quantified. To allow rapid strain differentiation in competition assays, these were carried out using pairs of strains in which one was GFP-labeled. We used the blue-light transilluminator Safe Imager 2.0 (Invitrogen) for visual discrimination. The log competitive index was obtained using the following formula: log (CFU_WT_/CFU_mutant_).

### Generation of transposon mutant libraries and sequencing

We constructed a transposon mutant library in *S. sonnei* strain 53G:LVP^Cm^ using transposomes prepared from mini-Tn5 and EZ-Tn5 Transposase (Epicentre). Transformants were collected from TSA plates containing 0.01% Congo Red, 8.5 μg/mL Cm, and 50 μg/mL Kn. The bacteria were resuspended at an OD_600_ of 100 and immediately stored in 1 mL aliquots at −80°C. The number of bacteria stored was retrospectively estimated by CFU determination.

Individual aliquots of the transposon library (10^9^ CFU/mL) were thawed and added to M9 and subjected to LTNS. After 18 days, cells were centrifuged (3,000 × *g* for 20 min at 4°C), and DNA was extracted from each LTNS culture pellet and from a frozen aliquot of the transposon library via phenol-chloroform extraction.

Two micrograms of DNA from each gDNA preparation were used to prepare TraDIS transposon-specific sequencing libraries following the protocol described in the TraDIS Toolkit method (85), with TraDIS adapter and primers as described previously (85). The resulting DNA was sequenced on an Illumina MiSeq platform using a MiSeq reagent kit version 2 (50 cycles) (Illumina, USA).

The analysis of TraDIS sequencing results was carried out using the Bio-TraDIS pipeline (https://github.com/sanger-pathogens/Bio-Tradis) as described previously (85). Processed reads were mapped to the reference genome (chromosome HE616528, plasmid A HE616529, plasmid B HE616530, plasmid C HE616531, and plasmid E HE616532). The input pool achieved read counts of 2,430,678 and 281,800 for the chromosome and large virulence plasmid, respectively. Statistical analysis was carried out using R version 3.2.3 included in the Bio-Tradis pipeline. The *bacteria_tradis* command was run using default settings, except the following parameters: --smalt_r 0 -m 0 and -t TAAGAGACAG. These settings enable mapping of multisite mapping reads, which would otherwise be filtered out, and ensure mapping of reads containing the expected transposon tag. The resulting insertion count plots (insert_site_plot.gz files, one per replicon, i.e., NC_016822, NC_016823, NC_016824, NC_016833, and NC_016834) were then processed with the tradis_gene_insert_sites command against the relevant annotated reference replicon. -trim3 was set to 0.1 to trim reads at the 3′ end, as many essential genes tolerate insertions toward the end of the coding sequence (86). A summary of genes that tolerated (i.e., non-essential genes) and did not tolerate (i.e., essential genes) insertions was produced by running the tradis_essentiality.R command on the resulting tradis_gene_insert_sites.csv files. Consensus essential genes were defined as genes where no insertions were identified in any of the samples. For the analysis of conditionally essential genes in long-term nutrient starvation, the logFC of read counts and the false discovery rate-adjusted *P*-value (*q*-value) for each gene were evaluated by using the tradis_comparisons.R script. Genes with significantly less transposon insertions in long-term nutrient starvation were selected by cutoff as logFC < - 2 and *q*-value ≤ 0.05.

### GO categories

Protein ANalysis THrough Evolutionary Relationships Classification System (https://pantherdb.org/) was used to classify genes that were identified by TraDIS according to their function using GO terms (GO Phylogenetic Annotation Project). Genes were attributed to significant functional categories (*P*-value < 0.05) using Fisher’s test and FDP correction.

### Mutagenesis and complementation

The plasmid vector pSEVA-612S served as a backbone for both mutagenesis and complementation constructs for the genetic modifications of the *S. sonnei* strain SS381.

The insertion of the GFP was made immediately downstream of *glmS*, previously shown to be a neutral site (49). For this purpose, the GFP-encoding gene was amplified from pULTRA-GFP and cloned into pSEVA-612S, flanked by ±500 bp upstream and downstream regions surrounding the insertion site downstream of *glmS*. Similarly, for the construction of deletion mutants, ±500 bp upstream and downstream regions flanking the target gene were cloned into plasmid pSEVA-612S. For the complementation of Δ*ldcA* and Δ*rseA* with *S. flexneri* alleles, *ldcA* and *rseA* were amplified from *S. flexneri* M90T and cloned into pSEVA-612S flanked by corresponding ±500 bp upstream and downstream regions of SS381. All PCRs were carried out using Q5 DNA polymerase (New England Biolabs, NEB) to ensure high-fidelity amplification, and cloning constructs were obtained by Gibson Assembly (NEB) or by restriction-ligation with T4 DNA ligase (NEB).

The resulting mutagenesis/complementation plasmids were then introduced into *E. coli* CC118λpir and then mobilized into *S. sonnei* SS381 by triparental conjugation with helper strain *E. coli* 1047 pRK2013. To resolve *S. sonnei* merodiploids, the I-SceI endonuclease encoded on plasmid pACBSR was induced by adding 0.4% (wt/vol) L-arabinose to the culture. After homologous recombination occurred, mutagenesis/complementation was confirmed by PCR screening and Sanger sequencing (Eurofins Genomics). Primers used in this study are listed in Table S6.

### Comparative sequence analysis

The comparison of essential genes between *S. sonnei* 53G and previously published essential gene data sets for Enterobacteriaceae was performed at the amino acid level with BLASTp (https://blast.ncbi.nlm.nih.gov/Blast.cgi). The *S. sonnei* 53G essential data set was used as query, and the EcoGene and ancestral essential data sets (47) were used as subjects. BLASTp searches were carried out using default parameters but with the following homology cutoff: query cover > 70% and amino acid identity > 40%.

To identify *S. sonnei*-specific variants potentially contributing to the differing survival between *S. sonnei* and *S. flexneri* strains, we compared the amino acid sequences of all conditionally essential genes identified by TraDIS in *S. sonnei* 53G with their counterparts in *S. flexneri* serotype 5a strain M90T and *S. flexneri* serotype 2a strain 2457T. Pairwise alignments were performed using BLASTp (with default parameters). The *S. sonnei* 53G protein sequences were used as queries, and the following reference genomes were used as subjects: *S. flexneri* serotype 5a strain M90T (NCBI RefSeq: NZ_CP037923.1), *S. flexneri* 2a strain 2457T (GenBank: AE014073.1), and *S. sonnei* strain 381 (GenBank: GCA_001248585.1). Genes were considered of interest if they were absent or non-homologous in both *S. flexneri* genomes and conserved (100% identical) between *S. sonnei* 53G and *S. sonnei* 381.

### Statistical analysis

Statistical analyses were conducted using GraphPad Prism (version 10.4.1). The specific tests applied to each data set are detailed in the corresponding figure legends. Statistical significance is denoted as follows: ns, not significant; *, *P* ≤ 0.05; **, *P* ≤ 0.01; *** *P* ≤ 0.001; and ****, *P* ≤ 0.0001.

## Data Availability

Transposon sequencing data are available for download from the Sequence Read Archive at NCBI (BioProject ID PRJNA1365785). All other data are included within the article and supplemental data files.

## SUPPLEMENTAL MATERIAL

Supplemental Material msystems.00088-26-s0001.pdf Supplemental methods and figures.

Table S1 msystems.00088-26-s0002.xlsx Essential genes.

Table S2 msystems.00088-26-s0003.xlsx Essential gene comparison.

Table S3 msystems.00088-26-s0004.xlsx TraDIS_CEG.

Table S4 msystems.00088-26-s0005.xlsx GO biological process.

Table S5 msystems.00088-26-s0006.xlsx BLASTp CEGs with *S. flexneri*.

Table S6 msystems.00088-26-s0007.xlsx Primers.

ASM does not own the copyrights to Supplemental Material that may be linked to, or accessed through, an article. The authors have granted ASM a non-exclusive, world-wide license to publish the Supplemental Material files. Please contact the corresponding author directly for reuse.
