# Supplementary material for: Genome-wide analysis exploring mechanisms used by Shigella sonnei to survive long-term nutrient starvation
Source: mSystems. 2026 Mar 16;11(4):e00088-26. doi: 10.1128/msystems.00088-26 (PMC13098226; doi:10.1128/msystems.00088-26)
Supplement: Supplemental Material — Supplemental methods and figures. [file msystems.00088-26-s0001.pdf]

Supplementary materials

Supplementary methodology

Bacterial growth kinetics

Overnight cultures of *S. sonnei* strains in TSB were diluted in fresh media to an OD_{600} of ~0.1. 100 µL of each suspension were added to a 96-well plate in triplicate. The plate was incubated at 37°C with the OD_{600} measured every 10 minutes over 12 hours with shaking (200rpm).

Protein sequence alignment

Multiple sequence alignments of amino acid sequences of interest were performed using Clustal Omega (https://www.ebi.ac.uk/jdispatcher/msa/clustalo) with default parameters.

Supplementary figures

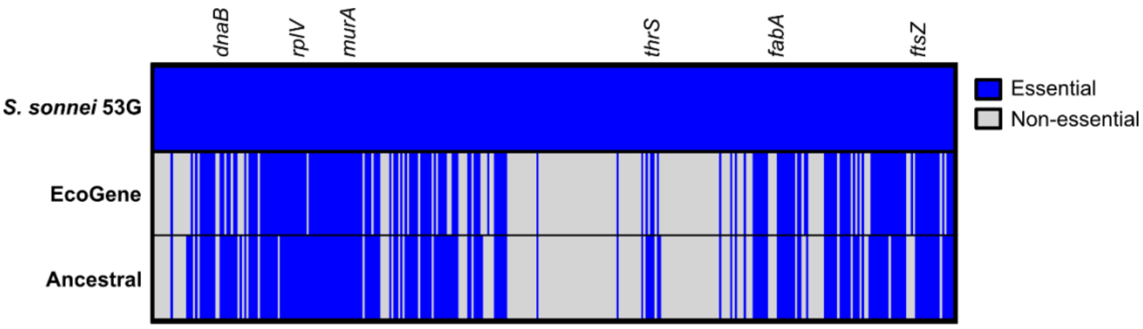

Supplementary Fig 1. Essential genes identified in *S. sonnei* 53G by TraDIS overlap with curated essential gene datasets in *Enterobacteriaceae*. Binary heatmap showing gene essentiality across *S. sonnei* 53G compared to the *Enterobacteriaceae* essentiality datasets EcoGene and Ancestral. Selected well-established essential genes are shown. Overlap of essential genes are indicated by colour, blue for essentiality and grey for non-essentiality.

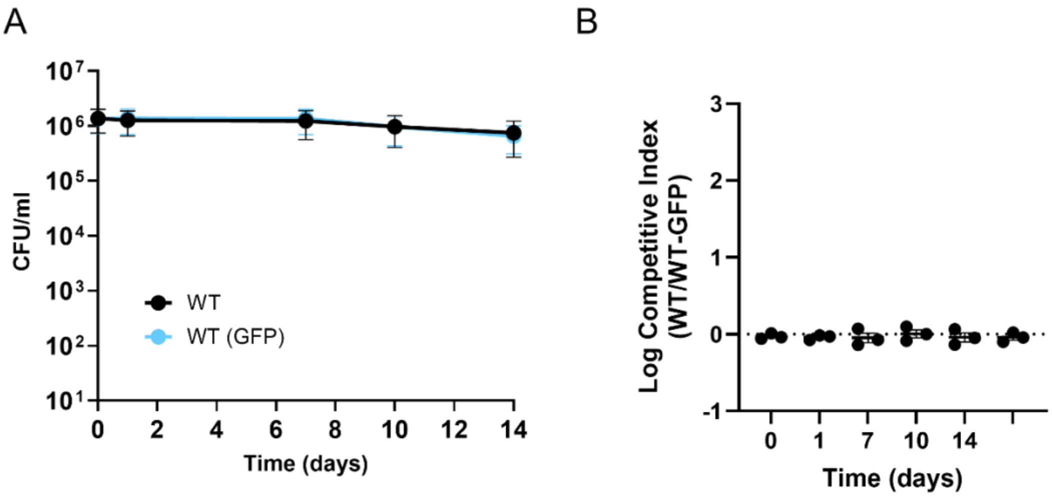

Supplementary Fig 2. Non-competitive and competitive LTNS survival of WT SS381 in comparison to SS381 (GFP). A) Non-competitive LTNS survival of WT SS381 and SS381 (GFP). Data show mean values ± SEM of n = 3 biological replicates. Log_{10}-transformed data was analysed by 2-way ANOVA with main column effect compared by Tukey's multiple comparison. ns = non-significant ($p > 0.05$). B) Competition assays between WT SS381 and SS381 (GFP). Logarithmic values of competitive index are plotted individually and as mean values ± SEM of n=3 biological replicates. The dotted line at log competitive index = 0 represents equal competition fitness. A one-way ANOVA with Dunnett's multiple comparisons was used to compare mean values at different time points relative to time 0. No significant differences were found.

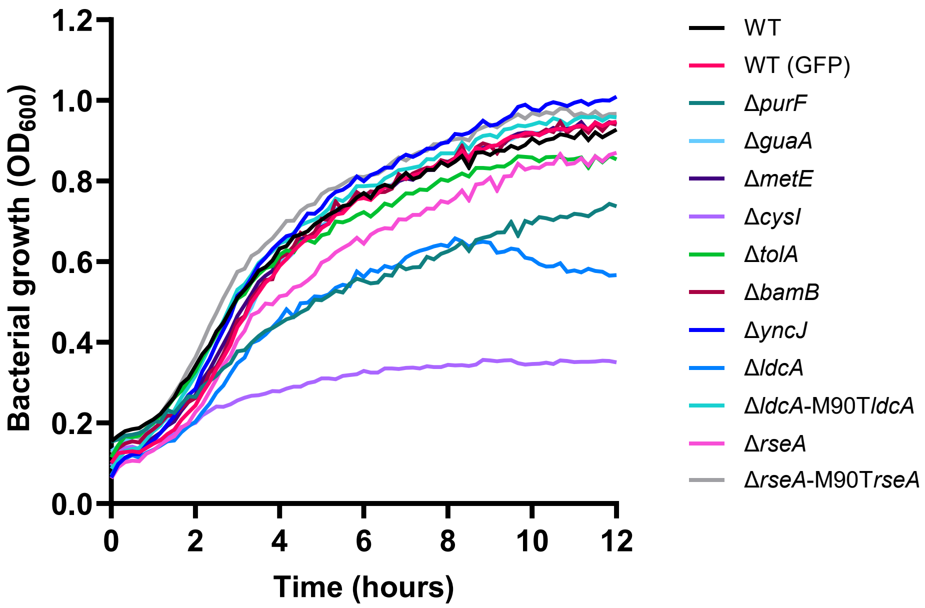

Supplementary Fig 3. Bacterial growth in nutrient rich (NR) conditions of strains used in this study. Growth was measured by recording optical density values at 600 nm (OD_{600}) every 10 minutes for a period of 12 hours in TSB at 37°C. Data show mean values of 2 biological repeats.

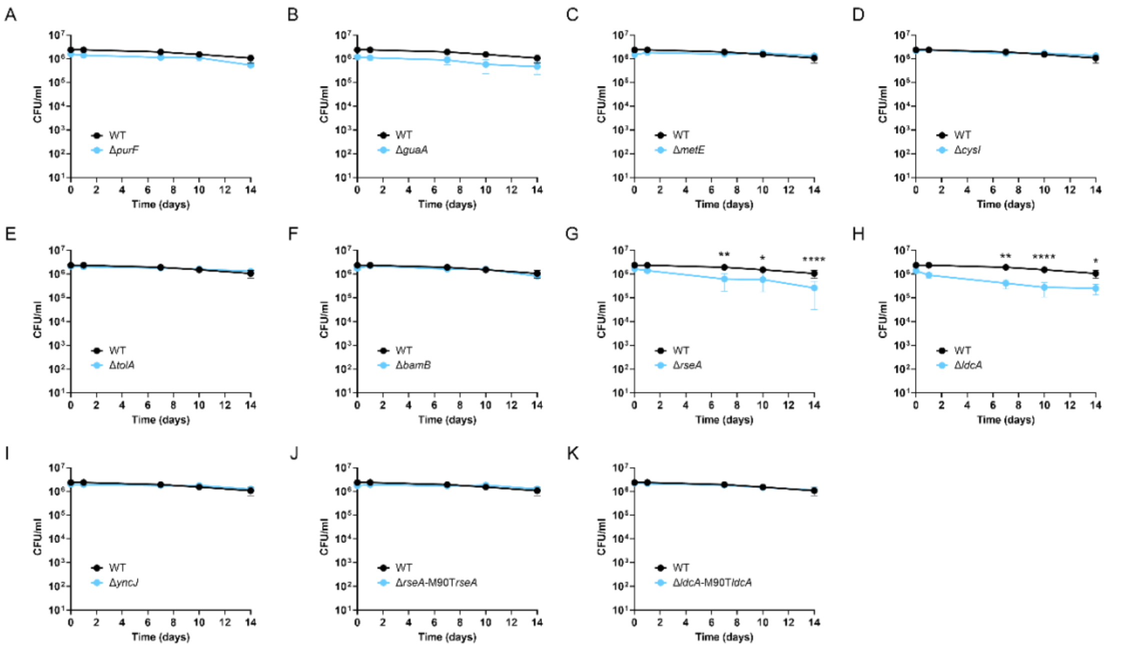

Supplementary Fig 4. Non-competitive LTNS survival of all mutants constructed for this study. Non-competitive LTNS survival of A) WT SS381 and SS381Δ*purF*, B) WT SS381 and SS381Δ*guaA,* C) WT SS381 and SS381Δ*metE,* D) WT SS381 and SS381Δ*cysI,* E) WT SS381 and SS381Δ*tolA,* F) WT SS381 and SS381Δ*bamB,* G) WT SS381 and SS381Δ*rseA,* H) WT SS381 and SS381Δ*ldcA,* I) WT SS381 and SS381Δ*yncJ,* J) WT SS381 and SS381Δ*rseA*-M90T*rseA,* K) SS381 and SS381Δ*ldcA*-M90T*ldcA*. For A-K data show mean values ± SEM of n = 3 biological replicates. Log_{10}-transformed data was analysed by 2-way ANOVA with main column effect compared by Tukey's multiple comparison. * = p ≤ 0.05, ** = p ≤ 0.01,**** = p ≤ 0.0001.

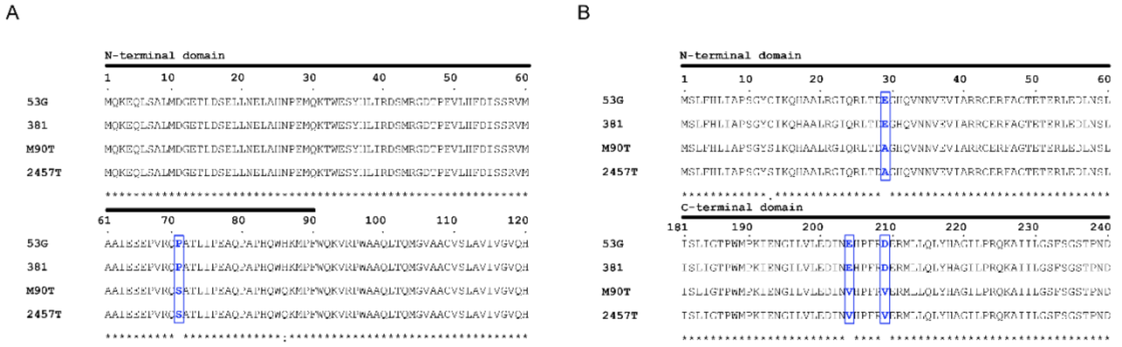

Supplementary Fig 5. Alignment of RseA and LdcA protein sequences from *S. sonnei* and *S. flexneri*. A) Amino acid alignment of RseA from *S. sonnei* 53G, *S. sonnei* 381, *S. flexneri* M90T, and *S. flexneri* 2457T showing one non-conservative (highlighted in blue) and located within the N-terminal σ^E-binding domain. B) Amino acid alignment of LdcA from *S. sonnei* 53G, *S. sonnei* 381, *S. flexneri* M90T, and *S. flexneri* 2457T showing three non-conservative amino acid differences (highlighted in blue). Alignments were performed using Clustal Omega.
